# Phenological shifts in lake stratification under climate change

**DOI:** 10.1038/s41467-021-22657-4

**Published:** 2021-04-19

**Authors:** R. Iestyn Woolway, Sapna Sharma, Gesa A. Weyhenmeyer, Andrey Debolskiy, Malgorzata Golub, Daniel Mercado-Bettín, Marjorie Perroud, Victor Stepanenko, Zeli Tan, Luke Grant, Robert Ladwig, Jorrit Mesman, Tadhg N. Moore, Tom Shatwell, Inne Vanderkelen, Jay A. Austin, Curtis L. DeGasperi, Martin Dokulil, Sofia La Fuente, Eleanor B. Mackay, S. Geoffrey Schladow, Shohei Watanabe, Rafael Marcé, Don C. Pierson, Wim Thiery, Eleanor Jennings

**Affiliations:** 1 Centre for Freshwater and Environmental Studies, Dundalk Institute of Technology, Dundalk, Ireland; 2 European Space Agency Climate Office, ECSAT, Didcot, Oxfordshire UK; 3 Department of Biology, York University, Toronto ON, Canada; 4 Department of Ecology and Genetics/Limnology, Uppsala University, Uppsala, Sweden; 5 Research Computing Center, Lomonosov Moscow State University, Moscow, Russian Federation; 6 Obukhov Institute of Atmospheric Physics, Russian Academy of Science, Moscow, Russian Federation; 7 Moscow Center of Fundamental and Applied Mathematics, Moscow, Russian Federation; 8 Catalan Institute for Water Research, Girona, Spain; 9 University of Girona, Girona, Spain; 10 University of Geneva, Institute for Environmental Sciences, Genève, Switzerland; 11 Pacific Northwest National Laboratory, Washington, USA; 12 Department of Hydrology and Hydraulic Engineering, Vrije Universiteit Brussel, Brussels, Belgium; 13 Center for Limnology, University of Wisconsin-Madison, Madison, WI USA; 14 Department F.A. Forel for Environmental and Aquatic Sciences and Institute for Environmental Sciences, University of Geneva, Geneva, Switzerland; 15 Department of Biological Sciences, Virginia Tech, Blacksburg, VA USA; 16 Department of Lake Research, Helmholtz Centre for Environmental Research-UFZ, Magdeburg, Germany; 17 Large Lakes Observatory and Department of Physics and Astronomy, University of Minnesota Duluth, Duluth, MN USA; 18 King County Water and Land Resources Division, Seattle, WA USA; 19 Research Department for Limnology, University of Innsbruck, Mondsee, Austria; 20 UK Centre for Ecology & Hydrology, Lancaster Environment Centre, Lancaster, UK; 21 Tahoe Environmental Research Center, University of California, Davis, CA USA

**Keywords:** Projection and prediction, Limnology

## Abstract

One of the most important physical characteristics driving lifecycle events in lakes is stratification. Already subtle variations in the timing of stratification onset and break-up (phenology) are known to have major ecological effects, mainly by determining the availability of light, nutrients, carbon and oxygen to organisms. Despite its ecological importance, historic and future global changes in stratification phenology are unknown. Here, we used a lake-climate model ensemble and long-term observational data, to investigate changes in lake stratification phenology across the Northern Hemisphere from 1901 to 2099. Under the high-greenhouse-gas-emission scenario, stratification will begin 22.0 ± 7.0 days earlier and end 11.3 ± 4.7 days later by the end of this century. It is very likely that this 33.3 ± 11.7 day prolongation in stratification will accelerate lake deoxygenation with subsequent effects on nutrient mineralization and phosphorus release from lake sediments. Further misalignment of lifecycle events, with possible irreversible changes for lake ecosystems, is also likely.

## Abstract (web summary)

Stratification has a considerable influence on lake ecology, but there is little understanding of past or future changes in its seasonality. Here, the authors use modelling and empirical data to determine that between 1901–2099, climate change causes stratification to start earlier and end later.

## Introduction

The vast majority of lakes worldwide are located in the Northern Hemisphere, notably north of the mid-temperate climate zone^1^. Lakes in these northern regions experience strong seasonality in climatic forcing, and, as a result, exhibit a pronounced seasonal variation in temperature^2^. Temperature has a fundamental effect on nearly all biological activities^3^. In lakes, temperature variations are complex, particularly where the phenomenon of thermal stratification adds an additional impact on biological productivity by directly influencing the size of the trophogenic zone in which photosynthesis takes place and also influencing nutrient supply from deep water^4–6^.

Seasonal stratification in lakes results from the interaction of turbulent mixing, forced mainly by surface wind-stress in conjunction with heat exchange at the air–water interface. During the spring–summer period, the stratifying effect of surface heat input competes with vertical mixing to determine the evolution of water column stability. In all but very shallow lakes (or some very deep lakes where stratification can be almost permanent), a stratified regime develops as heating outcompetes the effects of mixing—commonly referred to as the onset of stratification. This stratified regime continues until the autumn period, when lakes begin to lose heat to the atmosphere and both wind-stress and heat loss act to erode stratification and induce the autumnal overturn—also referred to as the break-up of stratification. The timing of stratification onset and break-up, collectively referred to as stratification phenology, plays a fundamental role in numerous physical, chemical, and biological lake processes, including population dynamics and interactions of aquatic species^7–10^.

Due to its ecological importance, changes in stratification phenology have been studied in individual lakes^11–13^ or a very few lakes within specific regions^14,15^. However, on a global scale, the influence of climate change on stratification phenology remains largely unexplored. This knowledge gap is of considerable concern given the high vulnerability of lake ecosystems, and the threatened biodiversity that they currently support, to climate change. To fill the fundamental knowledge gap, here we quantify past changes and assess future ones in lake stratification phenology across the Northern Hemisphere. We combined an analysis of long-term observational data from some of the best monitored lakes in the world, with daily simulations from an ensemble of lake models, each forced with climate data from an ensemble of twentieth and twenty-first century climate projections, under different anthropogenic greenhouse gas emission scenarios (Representative Concentration Pathway (RCP)): RCP 2.6 (low-emission scenario), 6.0 (medium–high-emission scenario), and 8.5 (high-emission scenario). Using all lake–climate model ensembles (see “Methods”), we simulated stratification phenology for Northern Hemisphere lakes from 1901 to 2099.

In this work, we show that climate change during the twenty-first century will have a considerable influence on stratification phenology, with lakes across the Northern Hemisphere stratifying sooner and maintaining their stratification for longer. In turn, our results show that the duration of the thermally stratified period will increase within a warming world, with lakes situated at higher latitudes projected to experience the greatest relative change.

## Results

### Stratification phenology in Northern Hemisphere lakes

**Fig. 1:**
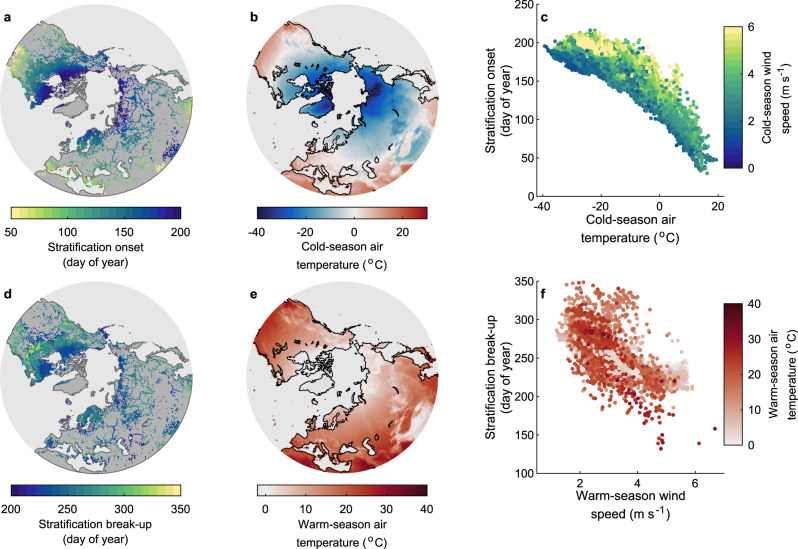
Simulations of lake stratification phenology across the Northern Hemisphere. Shown are the historic, averaged over all years from 1970 to 1999, spatial patterns in **a** the day of year of stratification onset, **b** the cold-season (November–April) average air temperature, and **c** the relationship between stratification onset, the cold–season air temperature, and wind speed. Also shown are the spatial patterns in **d** the day of year of stratification break-up, **e** the warm-season (May–October) average air temperature, and **f** the relationship between stratification break-up, the warm-season air temperature, and wind speed. All results are based on the average simulations across the lake–climate model ensemble.

**Fig. 2:**
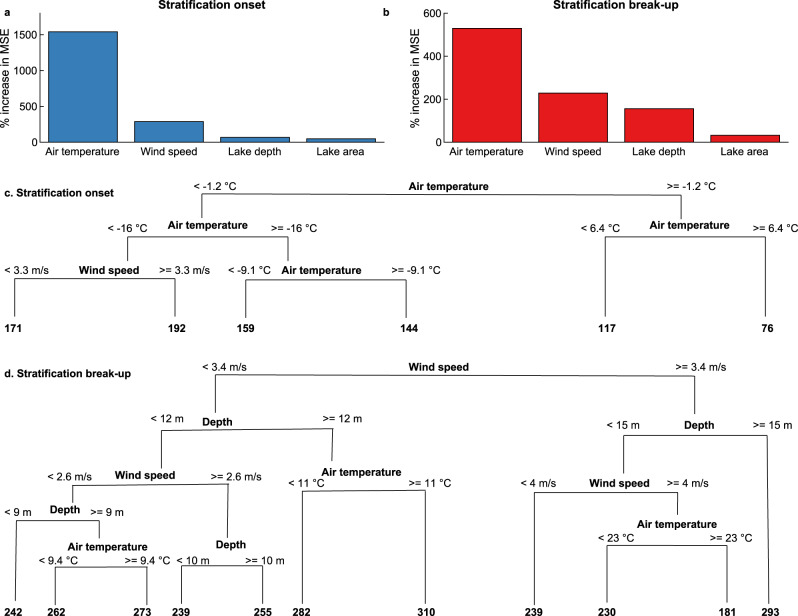
Climatic and in-lake drivers of lake stratification phenology. Importance of variables based on a random forest analysis in predicting **a** stratification onset and **b** stratification break-up. Variable importance is calculated as the percentage increase in mean square error (MSE) of predictions estimated with an out-of-bag coefficient of variation (CV) as a result of variables being permuted. Higher values indicate greater importance of a predictor variable to the set of decision trees. Regression tree showing the drivers of stratification onset date (**c**) and stratification break-up (**d**). The values below each of the leaves represent the date of stratification (shown as the day of year) on average for the lakes found within each group. All results are based on the average simulations across the lake–climate model ensemble. Cold season is defined as November–April and warm season is defined as May–October.

Our long-term daily simulations suggest that thermal stratification in Northern Hemisphere lakes during the historic period (averaged here for all years from 1970 to 1999) typically begins between March and July and ends between June and December (Fig. 1). Using a random forest model (see “Methods”), we investigated the climatic and lake morphological drivers of the simulated mean stratification onset and break-up dates. Predictor variables included seasonal (Northern Hemisphere cold and warm season) air temperature and wind speed, lake depth, and surface area (see “Methods”). Using these predictor variables, we were able to explain as much as 93% of the across lake variation in both the timing of historic stratification onset and break-up (Figs. 1 and 2a, b). For the timing of stratification onset, we found that the cold-season (November to April) air temperature was the most important driver, followed by the cold-season wind speed (Fig. 2a). The role of both air temperature and wind speed on the timing of stratification onset can be explained primarily due to their individual or, indeed, combined influence on lake surface temperature and, in turn, the vertical water density gradient in spring/summer. For example, as heat input acts to increase water column stability by warming the near-surface layer to above the temperature of maximum density (~3.98 °C), higher wind speed can both enhance heat loss via turbulent mixing^16^ and/or mix near-surface waters to greater depths, thus eroding any established vertical density gradient. Therefore, if air temperature is high and wind speed is low, one can expect an earlier onset of stratification. Conversely, if air temperature is low and wind speed is high, one can expect later stratification onset. In agreement with our expectations, a regression tree (which explained 85% of the variation in stratification onset) showed that lakes situated within a warmer climate stratified sooner than those elsewhere (*r* = −0.88; Supplementary Table 1a), and the latest stratification onset dates occurred in lakes located in the coldest and windiest regions (Fig. 2c). This result was confirmed by satellite-derived estimates of stratification onset for 60 Northern Hemisphere lakes (Supplementary Fig. 1; see “Methods”).

Lake morphological characteristics (depth and surface area) are also typically considered to be important factors influencing the timing of stratification onset in some lakes, due to the substantial period of convective mixing that typically occurs in large lakes prior to stratification onset in spring^17,18^. Specifically, in many large Northern Hemisphere lakes, winter lake surface temperatures are typically below the temperature of maximum density (e.g. during ice cover), resulting in an inversely stratified water column and the formation of a colder, less dense upper mixed layer above denser, warmer bottom waters^19^. Springtime heating leads to the loss of lake ice and warms near-surface waters, increasing water density and resulting in convective mixing through a deep volume of water with large thermal inertia. As a result, springtime heating can bring a more rapid rise in lake surface temperature in shallow lakes with smaller thermal inertia. Also, due to the higher surface momentum transfer from near-surface wind in large lakes^14^, winds can have a stronger influence on the date of stratification onset. However, across our studied lakes, the across-lake variations in air temperature and wind speed were the dominant drivers (Fig. 2a, c).

In contrast to stratification onset, wind speed (averaged over the warm season, May to October) was the most important driver of the mean stratification break-up date, followed by lake depth and warm season air temperatures (Fig. 2b). Lakes situated in regions with higher mean wind speeds experienced an earlier break-up of stratification (*r* = −0.81; Supplementary Table 1b). This result could, again, be verified by satellite-derived estimates of stratification break-up (Supplementary Fig. 1). Higher wind speeds can result in earlier stratification break-up by (i) enhancing turbulent heat loss at the air–water interface and (ii) mixing warm near-surface waters to greater depths and thus reducing the vertical density gradient. A regression tree analysis explained 80% of the variation in the timing of stratification break-up and revealed complex interactions between the warm season wind speed, air temperature, and lake depth in explaining stratification break-up across lakes (Fig. 2d). Stratification break-up was earliest in lakes shallower than 15 m located in warm and windy regions (Fig. 2d) and was latest in deep lakes located in cool, still regions. The depth effect of stratification break-up is primarily due to the presence of a deeper (and thus of higher volume) upper mixed layer in summer, resulting in lake temperatures being less sensitive to day-to-day changes in atmospheric forcing and thus experiencing a delayed break-up of stratification compared to shallower lakes^20^. In other words, larger lakes stay stratified for longer as both wind-stress and surface heat loss act on a larger volume of water, thus taking longer to erode stratification and induce overturn.

Our statistical model was able to explain over 90% of the variability in stratification phenology by a few globally available predictor variables (Fig. 2a, b). To explain the remaining variability, factors such as water transparency, which can differ across lakes due to changes in land management practices and precipitation patterns^21^, could potentially also be considered. Stronger light attenuation (i.e. due to darker surface waters) can increase lake surface water temperature in spring and summer, resulting in earlier stratification onset, but result in faster cooling in autumn^22–24^, thus leading to earlier stratification break-up. We also note that variations in salinity, which can vary among lakes due to natural process as well as human impact^25^, are not considered in our investigation. Changes in salinity, notably the within-lake vertical salinity gradient, could have an influence on stratification phenology by influencing the vertical density difference. In addition, water residence time is not considered in our study, which could influence the number of days in which a lake is continuously stratified and thus bias our estimates of stratification onset and break-up.

### Stratification phenology under climate change

**Fig. 3:**
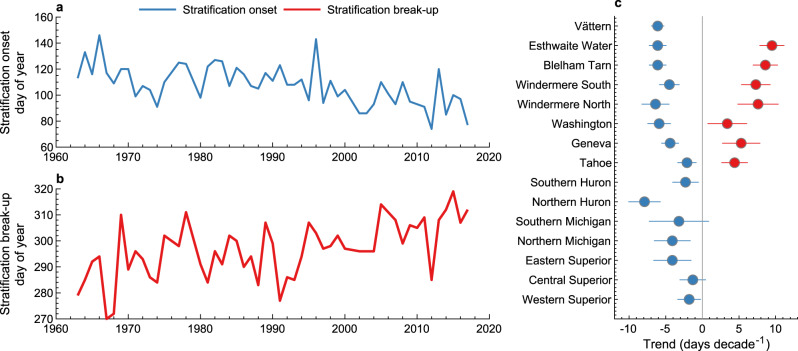
Observations of stratification phenology under historic to contemporary climate change. Shown are the temporal changes in stratification **a** onset and **b** break-up in Blelham Tarn, UK. Also shown, in **c**, are the calculated trends in stratification onset and break-up across all lakes with observational data investigated in this study (Supplementary Table 2). Note that stratification break-up data are not available for all lakes (see “Methods”), thus are not shown in **c**. Error bars in **c** represent the standard error of the calculated trend.

Our model projections during the historic period, from 1901 to 2005, show noticeable temporal changes in lake stratification phenology, particularly since the 1980s (Supplementary Fig. 2). Monitoring data from lakes across the Northern Hemisphere (see “Methods”) confirm such changes (Fig. 3 and Supplementary Figs. 3 and 4). For example, Lake Tahoe (California, Nevada) has experienced a considerable change in stratification phenology since the 1960s, with lake stratification onset occurring 2.1 days earlier and stratification break-up occurring 4.4 days later per decade. Similar long-term changes are observed in the United Kingdom with, for example, Blelham Tarn (English Lake District; Fig. 3) now stratifying 24 days earlier and maintaining its stratification for an additional 18 days, compared to the start of the observational record (here we compare averages between the first [1963–1972] and last [2008–2017] 10 years of observational data available). The upper North American Great Lakes (Superior, Huron, Michigan), which represent some of the world’s largest freshwater ecosystems and contain irreplaceable biodiversity, are also experiencing rapid changes in stratification phenology. For example, from 1980 to 2019 stratification onset has changed at an average rate of 3.5 ± 2.2 days per decade (quoted uncertainties represent the standard deviation across the Great Lakes) and can even vary by up to 48 days between some extreme years (Supplementary Fig. 5).

During the twenty-first century (2006 to 2099), air temperature is projected to increase considerably across the Northern Hemisphere land surface (Supplementary Fig. 6). Specifically, climate model projections suggest that by 2070–2099 the cold-season air temperature will increase, relative to 1970–1999 (hereafter all future changes are quoted relative to this base-period average), by an average of 2.8 ± 0.9 °C, 5.0 ± 1.4 °C, and 7.5 ± 1.9 °C between RCP 2.6, 6.0, and 8.5, respectively (quoted uncertainties represent the standard deviation from the model ensemble). Similar changes are also projected during the warm season, with air temperatures increasing by 2.1 ± 0.8 °C, 3.8 ± 1.1 °C, and 5.8 ± 1.6 °C between RCP 2.6, 6.0, and 8.5, respectively. In comparison to these projected changes in air temperature, the projected change in near-surface wind speed is relatively minor. Notably, across the Northern Hemisphere land surface, cold-season wind speed is projected to change by −0.03 ± 0.05 ms^−1^, −0.03 ± 0.07 ms^−1^, and −0.03 ± 0.09 ms^−1^ between RCP 2.6, 6.0, and 8.5, respectively. During the warm season, near-surface wind speed will change by −0.1 ± 0.06 ms^−1^, −0.1 ± 0.08 ms^−1^, and −0.14 ± 0.1 ms^−1^ between RCP 2.6, 6.0, and 8.5, respectively. However, as momentum and mechanical energy fluxes across the air–water interface scale as the wind speed squared and cubed, respectively^26^, modest fractional changes in wind speed may cause substantial change in stratification dynamics^27^. Therefore, while the projected changes in wind speed, when averaged across the Northern Hemisphere land surface, are relatively small and cannot, with confidence, be described as either decreasing or increasing under all RCPs, local-scale projected changes in wind speed (Supplementary Fig. 6) could have a considerable influence on stratification phenology this century.

**Fig. 4:**
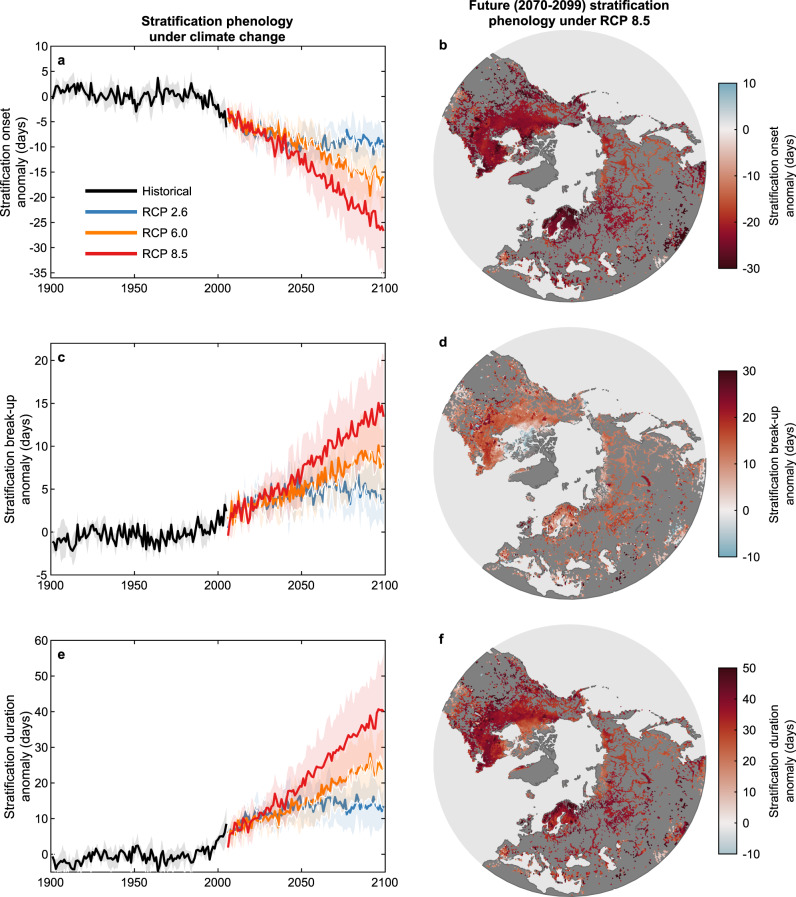
Historic and future projections of lake stratification phenology. Temporal and spatial variations in **a**, **b** the onset and **c**, **d** the break-up of thermal stratification, as well as **e**, **f** the duration of the thermally stratified period. The temporal changes in lake stratification phenology are shown **a**, **c**, **e** from 1901 to 2099 under historic and future climate forcing (RCP 2.6, 6.0, 8.5). The thick lines show the average across all lake–climate models, and the shaded regions represent the standard deviation across the model ensemble. **b**, **d**, **f** show the spatial patterns in stratification phenology anomalies by the end of the twenty-first century (averaged over all years from 2070 to 2099) under RCP 8.5. Anomalies are quoted relative to the 1970–1999 base-period average.

In line with the projected changes in climatic forcing, our lake model simulations suggest that lake stratification phenology will change substantially in the near future (Fig. 4). Under RCP 2.6, the onset of stratification will occur 8.8 ± 3.5 days earlier and the break-up of stratification will occur 4.2 ± 2.6 days later by the end of the twenty-first century (averaged over all years and lakes from 2070 to 2099). Under RCP 6.0, stratification onset and break-up will occur 14.7 ± 5.1 days earlier and 7.3 ± 3.8 days later, respectively. The largest change in stratification phenology are projected under RCP 8.5 with stratification onset and break-up, respectively, occurring 22.0 ± 7.0 days earlier and 11.3 ± 4.7 days later, on average across the Northern Hemisphere. To investigate further these long-term projected changes in stratification phenology and to explore their regional variability, we separate the studied lakes according to the thermal regions in which they reside (see “Methods”). Approximately 98% of our studied lakes are situated within the four northernmost thermal regions (Supplementary Fig. 7a, b): Northern Frigid (31%), Northern Cool (43%), Northern Temperate (16%), and Northern Warm (8%). Within these four regions, our simulations suggest that the change in stratification onset by 2070–2099 will be relatively similar (Supplementary Fig. 7c–e). For example, under RCP 8.5, the average projected change in stratification onset across the four thermal regions are Northern Frigid: Northern Cool: Northern Temperate: Northern Warm = −21.6 ± 4.9:−22.5 ± 3.7:−22.8 ± 5.7:−21.8 ± 7.3 days. The average change in stratification break-up are 10.2 ± 4.8:12.5 ± 4.0:13.4 ± 5.5:11.6 ± 8.2 days, respectively.

**Fig. 5:**
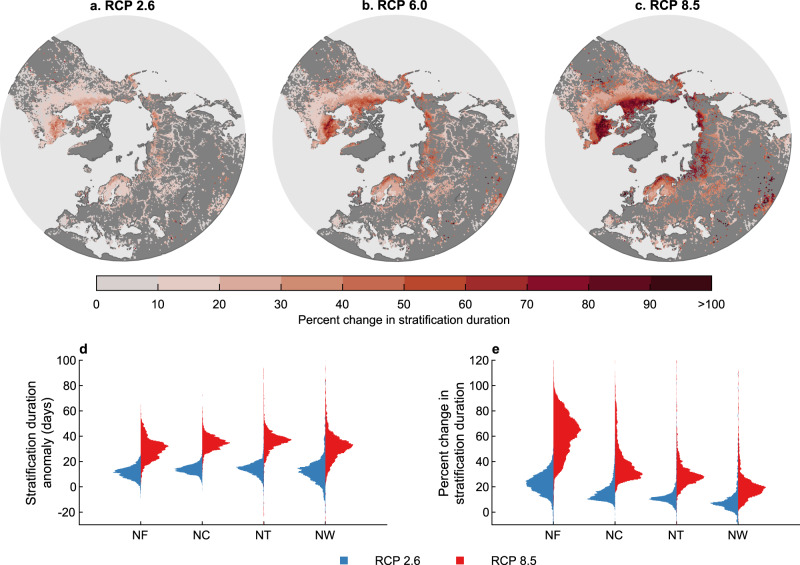
Absolute and percentage change in the duration of stratification during the twenty-first century. Shown are the spatial patterns in the percentage change in the duration of stratification by the end of the twenty-first century (2070–2099), relative to 1970–1999, under **a** RCP 2.6, **b** RCP 6.0, and **c** RCP 8.5. Also shown, across the four dominant Northern Hemisphere lake thermal regions, are **d** changes in stratification duration and **e** the percentage difference by the end of the century under RCP 2.6 and RCP 8.5. The thermal regions defined include Northern Frigid (NF), Northern Cool (NC), Northern Temperate (NT), and Northern Warm (NW). The distribution of these thermal regions across the Northern Hemisphere are shown in Supplementary Fig. 7.

Given the projected change in both stratification onset and break-up by the end of this century, the duration of the stratified period will increase (Fig. 4e, f). Under RCP 2.6, 6.0, and 8.5, the duration of stratification will increase by 13.0 ± 6.0, 21.9 ± 8.5, and 33.2 ± 11.6 days, respectively, on average. The magnitude of change in the duration of stratification differs slightly across the lake thermal regions (Fig. 5). For example, under RCP 8.5, the average change in stratification duration by 2070–2099 across the thermal regions are Northern Frigid: Northern Cool: Northern Temperate: Northern Warm = 29.9 ± 8.4:34.8 ± 5.7:35.7± 11.6:31.9 ± 13.9 days. However, while the magnitude of change will be comparable, the percentage change in stratification duration will be considerably greater in the northernmost thermal region i.e. Northern Frigid (Fig. 5). Under RCP 8.5, the percentage increase in the duration of stratification across the thermal regions are Northern Frigid: Northern Cool: Northern Temperate: Northern Warm = 61.4 ± 15.9%:39.0 ± 15.2%:29.3 ± 11.6%:20.5 ± 12.5% (Fig. 5). Thus, a similar average increase of ~30 days in stratification duration projected in the Northern Frigid and Northern Warm thermal regions correspond to an almost threefold difference in the relative change in stratification duration. This is due to a shorter mean duration of thermal stratification during the historic period at higher latitudes, compared to lakes situated in the warmer southern regions.

The severity of change in stratification phenology by the end of the twenty-first century is explained primarily by the magnitude of change in the climatic drivers investigated. As expected, regions which are projected to experience the greatest change in cold- or warm-season air temperature and wind speed are also expected to experience the greatest change in stratification phenology. We also find a strong relationship between the anomalies in the day of year of ice break-up and the change in the day of year in which lakes stratify during the twentieth and twenty-first centuries (Supplementary Fig. 8), with later ice cover break-up intuitively leading to a later onset of thermal stratification. Furthermore, regions that are projected to experience an increase in near-surface wind speed by the end of the twenty-first century should expect lower changes in stratification phenology compared to lakes in other regions, with increasing wind speeds counteracting some of the global warming-induced changes in stratification phenology. On the contrary, a decline in near-surface wind speed by the end of the century will likely exacerbate the influence of climatic warming on lake stratification in Northern Hemisphere lakes^27^.

## Discussion

We expect that the changes in stratification phenology that we have described here will have far reaching implications for lake ecosystems throughout the entire twenty-first century. For example, an earlier onset of stratification, as our historic and future simulations suggest under climate change, has been found to facilitate phytoplankton growth in lakes through the amelioration of light limitation. In a number of lakes, this has brought forward the onset of the spring bloom^7,9,28^. The earlier growth of phytoplankton communities has the potential to alter the species composition and succession, where increased abundances of early-season taxa or cold-adapted species are favoured^29,30^. Earlier growth may also open up ecological niches later in the spring that enable the growth of potentially harmful filamentous species^29^ that may represent a poorer food resource for grazers. The extent to which trophic mismatching is likely to occur between peaks in phytoplankton and zooplankton grazers is unclear, since changes in peak timing in resource and consumer differ between studies^8,28,29^, although rates of change at higher trophic levels may be slower^28^, with potential for impacts on fish recruitment and survival^31^. The extent to which changes in stratification phenology will influence lake ecosystems will depend on a suite of variables, including trophic interactions and community dynamics, but could potentially lead to abrupt shifts in global lake ecosystems during the current century^8,9,28^.

The change in stratification phenology that our ensemble projections suggest are also very likely to have a considerable influence on the interactions between surface and bottom waters, such as the exchange of oxygen, nutrients, and carbon. A longer decoupling between epilimnetic and hypolimnetic ecosystem processes will influence some of the key rules of life in lake ecosystems to which temperature, light, nutrient regimes, and habitat succession belong^32^. The severity of such impact is also, however, dependent on limiting conditions in a particular lake. For example, an increasing duration of thermal stratification, closely coupled to oxygen concentrations in bottom waters^33^, facilitates the process of oxygen depletion, by preventing vertical mixing of oxygen from the surface. We suggest that most pronounced ecosystem changes will occur in lakes that will experience hypoxic conditions for the first time and in lakes where the duration of hypoxia will be extended as a response to the projected prolongation in stratification. Generally, it is expected that the projected prolongation in stratification will result in the deoxygenation of productive lakes^34^, where, in the worst case, the occurrence of fish die-off events will increase^35^. Anoxic conditions at the sediment–water interface have the potential to result in substantial nutrient leakage from sediments^34^ and to have subsequent effects on nutrient mineralization. In particular, declines in redox potential in productive lakes can reduce iron and allow for a substantial phosphorus release from the sediments to the overlying water^36^. Anoxia is also known to favour the production of the potent greenhouse gas methane, which is produced in and emitted from lakes at globally significant rates^37,38^. Thus, the changes in stratification phenology that we have described here, and the resulting decrease in bottom water oxygen concentrations that could occur in many lakes, could contribute to a further acceleration of climate change and thus represents a positive feedback that should be carefully considered in future climate change impact studies. However, it is also important to consider that an earlier onset of stratification can also imply a shorter ice-cover season within a warming climate (Supplementary Fig. 8), and thus less winter hypoxia^39^. A decline in winter hypoxia could consequently lead to less greenhouse gas accumulation during winter.

Many of the ecosystem services that lakes provide, ranging from the delivery of drinking water and food to recreation, may be endangered by the projected change in stratification phenology during the twenty-first century, particularly in urbanized and agricultural regions where lakes are already eutrophic. In these regions, a prolonging of lake stratification has been shown to increase the occurrence and intensity of toxic algal blooms^40,41^, which, as well as influencing the aesthetic appeal of lakes, can lead to mass mortalities of fish and birds, as well as provide a serious health threat for cattle, pets, and humans. Algal blooms rank among some of the main causes of poor water quality issues in lakes and, with the projected increase in water scarcity during the twenty-first century, will become an increasingly important issue in the future as freshwater demands exceed its regional availability. When combined with the host of other human effects on lakes, such as eutrophication^10^, phenological changes in stratification will very likely become a source of additional ecological stress. Immediate actions, such as not only reducing global warming but also mitigating nutrient loads to lakes, are needed to counteract the effects of ongoing and projected fast phenological changes to sustain ecosystem services from lakes worldwide.

## Methods

### Simulated lake stratification phenology

There is no universal definition of stratification in lakes, but water density thresholds are often used^42,43^. In this investigation, the onset and break-up of stratification were calculated from simulated lake temperature data (see below) and defined as the first and last day of year, respectively, in which a temperature-driven density difference of 0.1 kg m^−3^ existed between surface and bottom waters. Other density thresholds could also have been used, which could influence the derived stratification metrics. However, in this study we follow the Inter-Sectoral Impact Model Intercomparison Project (ISIMIP) lake sector protocol and define stratified conditions based on the aforementioned vertical density difference. Furthermore, the onset and break-up of thermal stratification were identified as the start and end date of the longest thermally stratified period in each studied lake—thus ignoring intermittent stratified events during the transitional periods of summer stratification. Furthermore, the start and end date of stratification was only investigated for each 0.5° grid where the seasonal mixing regime resembled that of monomictic or dimictic lakes. These lake mixing regimes were defined following the same procedure as ref. ^44^ to simulate the mixing regime of lakes worldwide. Dimictic lakes are those that experience two vertical mixing events per year (i.e. during spring and autumn following the inversely stratified period in winter and the positively stratified period in summer), and monomictic lakes are those that experience one full vertical mixing event (i.e. vertically mix during the autumn following the summer stratified period and usually do not inversely stratify in winter).

### Global lake simulations

Lake temperature data used in this study were simulated, within the ISIMIP phase 2b (ISIMIP2b) lake sector, with a suite of independently developed lake models: ALBM, GOTM, LAKE, and SimStrat-UoG. Following the ISIMIP2b global lake sector protocol, each of these lake models were used to simulate vertical lake temperature profiles at a 0.5°-by-0.5° grid resolution, based on the mean depth and surface area of all lakes within a given 0.5° grid (i.e. the average depth of all known lakes and the surface area covered). The locations, depths, and grid-scale fractions of lakes within each 0.5° grid were determined by the Global Lake Data Base version 1 (refs. ^45–47^). Lake information was aggregated from the original 30 arc sec resolution to a 0.5°-by-0.5° grid lake depth field.

To drive each lake model, bias-corrected climate model projections from ISIMIP2b were used, specifically projections from GFDL-ESM2M, HadGEM2-ES, IPSL-CM5A-LR, and MIROC5 for historic (1901–2005) and future periods (2006–2099) under three scenarios: RCP 2.6, 6.0, and 8.5. These pathways encompass a range of potential future global radiative forcing from anthropogenic greenhouse gases and aerosols, and results span a range of potential impacts on lake temperature and stratification. The data used to drive each lake model included projections of air temperature at 2 m, wind speed at 10 m, surface solar and thermal radiation, and specific humidity, which were available at a daily resolution. These data were used as inputs to the model after bias adjustment to the EWEMBI reference data set^48,49^.

### Statistical methods

We conducted random forests analysis to identify the climatic and lake morphological drivers of the mean stratification onset and break-up dates during the historic period. The randomForest function in R^50,51^ was used for this analysis. Random forests are based on an ensemble of decision trees^52^. We generated 1000 trees from which we calculated variable importance to generally identify how often a predictor variable was the most important predictor in a single decision tree. We used the mean decrease in accuracy, describing the prediction error calculated by the mean squared error on the out-of-bag portion of the data^50^. Spearman’s correlations were subsequently calculated to assess the relationships between all variables using the correlate function in R^53^.

The random forest and Spearman correlation analyses suggested that there may be interactions between predictor variables, thus precipitating the use of regression trees to further understand the drivers of stratification phenology. Regression tree models are able to effectively incorporate non-linear relationships, multicollinearity, and interactions between predictor variables^54^. We used the climatological mean day of stratification onset and break-up as response variables. Predictor variables included seasonal air temperatures and wind speed, lake depth, and lake surface area. Seasonal climate data were used to explain the across-lake variations in stratification phenology in order to (i) ensure consistency across the studied lakes and (ii) to capture both direct and indirect (e.g. the influence of ice cover on stratification onset) climatic effects. However, stratification phenology in an individual lake will be influenced most strongly by climatic factors during the preceding weeks to months of the mean onset/break-up dates. The most parsimonious regression tree was selected by pruning the tree to the level where the complexity parameter minimized the cross-validation error. We calculated the percentage variation explained by the regression tree (*R*^2^) as: *R*^2^ = 1 − relative error^55^. Regression trees were developed in R using the rpart and rpart.plot functions^56,57^.

To investigate the regional variability in lake stratification responses to climate change, we separated the studied lakes into the thermal regions in which they are located, following the definitions of ref. ^2^. Five lake thermal regions were identified in the Northern Hemisphere. The vast majority (98%) of the studied lakes were located in the four northern most thermal regions, and thus we only explored the regional variability in these lakes.

The trends in both onset and break-up of observed stratification (see below) were assessed in R^51^ using either a generalized additive modelling (with a cubic regression spline using cross-validation to optimize *k*, the number of knots)^58^ or a linear model. The optimum model selected based on the Akaike information criterion. The residuals from each model were checked for any breach of assumptions. A correlation structure was added to the model for Windermere South Basin for stratification break-up to account for autocorrelation in model residuals.

### In situ lake stratification phenology

Observational data from some of the best-monitored lakes in the world were used in this study to investigate the long-term changes in lake stratification phenology and to complement our findings from the multi-model simulations. Each lake data set used in this study are described briefly below.

#### Lake Washington, USA

Lake Washington is a relatively large (surface area of 87.6 km^2^) and deep (maximum depth of 65.2 m) monomictic lake, with temperature profiles having been collected weekly to twice monthly by the University of Washington at a central site within the lake since 1963. In this study, the lake temperature profiles were first interpolated to uniform 1-m depth intervals and then combined and interpolated to a uniform daily resolution. The interpolated data were then exported into a format suitable for use in rLakeAnalyzer^59^. Functions in rLakeAnalyzer were used to determine the density difference in the surface 1 m and near bottom water (55 m) each day over the period of the record. The onset and break-up of seasonal stratification were determined, similar to those from the ISIMIP2b simulations, by finding the first and last day of year in which a density difference of >0.1 kg m^-3^ existed (stratification phenology in all lakes with in situ data, described hereafter, were defined relative to this threshold).

#### Lake Tahoe, USA

Lake Tahoe is a large (surface area of 495 km^2^) and deep (maximum depth of 501 m) sub-alpine, monomictic lake. Routine temperature measurements have been taken at a near-shore site (Index, 160 m deep) and an off-shore site (Mid-lake, 500 m deep) since 1970 by the Tahoe Environmental Research Center at the University of California, Davis. The Index site has been sampled approximately every 12 days and Mid-lake every 29 days. The surface water temperature measured at the two stations were combined and then linearly interpolated to obtain values at daily resolution. Deep water (400 m) temperatures from Mid-lake station were also linearly interpolated.

#### Lake Geneva, France/Switzerland

Lake Geneva is a deep (maximum depth 308 m) perialpine lake with a surface area of 580 km^2^. Temperature profiles have been recorded at the deepest point of the lake since 1957 by OLA-IS, AnaEE-France, and INRAE, at bi-weekly to monthly frequency. To determine onset and break-up of stratification, we compared the surface water density (i.e. observation closest to the lake surface) with the average water density between 40 and 70 m, the depth at which the lake stratifies seasonally each year^60^. Stratification phenology was not calculated for years in which the interval between subsequent observations was >30 days during the period of stratification onset and break-up.

#### English Lake District, UK

Data from the UKCEH Cumbrian Lakes Monitoring Programme were obtained for the four longest running time series on Blelham Tarn (BT; 1963–2017), Esthwaite Water (EW; 1957–2017), and the north (NBW; 1951–2017) and south basins (SBW; 1967–2017) of Windermere. Weekly or fortnightly temperature profile data from each lake was visually examined and years were excluded where there was insufficient profile data during the months around the onset and end of the main stratified period in summer (BT = 1, NBW = 7, SBW = 1). In addition, temperature profiles (*n* = 7) from individual dates were removed from the whole data set where there was instrument error reported or where profiles were poorly resolved due to missing data. Water temperature data from each lake was then linearly interpolated onto a daily time step and regular depth resolution of 1 m using the interp function from the akima package in R^61^. The interpolated water temperature data was converted to water density using rLakeAnalyzer^59^, and the difference between top and bottom densities was calculated. All stratified periods in each year, for each lake, were identified, with the start day of the main summer stratified period considered to be the latest day before day of year 200 where a stratified period had a duration >14 days, and the end day was defined as the final day of stratification for that stratified period.

#### North American Laurentian Great Lakes

Long-term full water column temperature data are scarce in the Laurentian Great Lakes. The longest continuous moored record has been collected by the Great Lakes Environmental Research Laboratory, which has had a site occupied in southern Lake Michigan since 1991. Another site in western Lake Superior has been occupied since 2005 (ref. ^62^). Neither of these have sufficient data to explore climate-related changes in stratification phenology. However, there are several sites at which surface water temperature has been measured by meteorology buoys in recent decades. Surface water temperature from these buoys can be a useful proxy for stratification phenology^11^. Specifically, when the surface water temperature reaches the temperature of maximum density (3.98 °C at the surface of fresh water lakes), stability considerations require that deep water temperatures must be between the temperature of maximum density for a given depth (this temperature decreases by 0.2 °C for every 100 m depth) and the surface temperature of maximum density. Therefore, the heat content of the water column is known to a high level of confidence, so that the date of this occurrence is a useful phenological indicator. Reaching 3.98 °C at the surface in the spring thus coincides with the onset of positive stratification^63^. In this study, we investigate stratification phenology in three of the Laurentian Great Lakes—Superior, Michigan, and Huron with sub-daily surface temperature data from surface meteorology buoys, which have been deployed by the National Data Buoy Center (https://www.ndbc.noaa.gov) in the open (offshore) waters of the lakes since 1980 (although not all years had suitable data). Ontario is excluded from the study since data are only available as far back as 2002; Erie is excluded because its surface temperature is typically already above the temperature of maximum density when the buoys are deployed in the spring. At all sites, the buoys are typically recovered before the lake cools through the temperature of maximum density, so there is not sufficient data to examine the break-up of stratification. In these lakes, we determine stratification onset as the first day of year on which the minimum lake surface temperature exceeds 3.98 °C.

#### Vättern, Sweden

No depth-resolved water temperature data are available for Vättern, the second largest lake by surface area in Sweden. However, long-term daily surface temperature observations, measured at a drinking water intake point in the north-eastern part of the lake, are available since 1955. These data (1955–2017) were published previously by ref. ^64^ and were updated in this study to 2019. Stratification onset was estimated in Vättern, following the water temperature–density considerations described for the Laurentian Great Lakes, as the first day of year in which the surface water temperature increased above 3.98 °C.

### Satellite-derived stratification phenology

As described above, surface temperature observations can be used as a proxy for lake stratification phenology. Specifically, by identifying the day of year in which lake surface temperatures cross a 3.98 °C threshold, surface temperatures can provide a useful phenological indicator of stratification^63^. Here, the across-lake variability in stratification phenology, as calculated from the model ensembles during the historic period, were compared with those estimated from satellite-derived lake temperatures. Notably, stratification phenology was estimated using lake surface temperatures from the European Space Agency’s Climate Change Initiative (CCI) Lakes project (CCI Lakes; http://cci.esa.int/lakes), which contains daily observations from 250 lakes worldwide. From these lakes, we selected those situated in the Northern Hemisphere (>30°N) and those large enough for the existence of a 10 × 10 pixel array of pure water surrounding the lake centre, defined as the maximum distance to land, following the recommendations of ref. ^65^ for minimizing land contamination of the satellite retrievals.

Not all lakes within the CCI Lakes data set were suitable for this study. Here, we were only interested in freshwater lakes (due to the temperature–density relationship described previously that complicates the identification of stratification from surface observations), and thus we removed all brackish/saline lakes from the analysis. To identify which lakes were freshwater and which were brackish/saline, we first extracted the names of each lake within CCI Lakes, achieved by mapping the CCI lake mask onto the Hydrolakes data set^66^. Lake names were then used within a literature search to identify whether a given lake was freshwater/saline/brackish. For lakes with no available information, we then used the Hydrolakes database to identify which lakes were likely situated in terminal sinks (i.e. with no throughflow) and were thus likely to be brackish/saline. To identify these lakes, we used the average discharge information available in Hydrolakes, and all lakes with negligible discharge (<1 m^3^ s^−1^) were removed from the analysis.

Another criterion for estimating stratification phenology from surface temperature observations is that the studied lakes must experience seasonal stratification, notably that they are characterized as monomictic or dimictic. For example, the 3.98 °C temperature threshold would not be an indicator of stratification phenology in polymictic lakes (i.e. those that mix frequently). To identify the mixing regime of each lake, we again used information available in the published literature. For lakes with no information available, we used modelled lake surface and bottom water temperatures to simulate the lake mixing regime following the methods of ref. ^44^. Lake surface and bottom water temperature simulations for each lake were extracted from ECMWF’s ERA5 product^67^ for the corresponding grid (0.25° resolution) of each lake centre pixel. ERA5 lake surface temperatures have been shown previously to accurately simulate the thermal environment of lakes at a global scale^68^.

For all lakes that were considered suitable to estimate stratification phenology from surface temperature observations, notably those that passed the criteria described above, we calculated the lake mean surface temperature time series from the satellite data^17^, which were then used to identify the first and last day of year in which surface temperature cross the 3.98 °C threshold. Only observations from 2007 to 2019, the time period in which most satellite retrievals were available in ESA CCI Lakes v1.0, were used in this study, as the low temporal resolution of retrievals prior to 2007 could bias the calculated stratification phenology. Furthermore, the satellite data were only used to identify the onset and break-up of thermal stratification if the temporal resolution of observations were considered adequate for each year. Specifically, we only used the satellite observations to estimate stratification onset if measurements were available at a temporal frequency of <5 days for at least 75% of the ice-free period, defined using the lake ice cover data from ESA CCI Lakes. Linear interpolation was then used to generate a gap-free time series when each of the previous criteria were met. The onset and break-up of thermal stratification averaged across all years per lake were then compared to the cold- and warm-season climatic data (air temperature and wind speed), respectively. For this comparison, we used climate data from ERA5, extracted from the same lake centre pixels as the lake temperatures described above. ERA5 data were used, as opposed to the ISIMIP2b historic climate projections, because the latter finish in 2005, and the satellite data used do not start until 2007. This also forms an additional validation of the relationships shown between climate and lake stratification, as both independent data sets produced similar results. One additional limitation for this criterion for defining stratification break-up is in shallower dimictic or monomictic lakes, which often mix at significantly higher temperatures than 3.98 °C due to the build-up of heat within the hypolimnion during summer.

## Supplementary information

Supplementary Information

## Data Availability

All lake model simulations are available at https://esg.pik-potsdam.de. Lake surface water temperature and lake ice cover data are available from ESA CCI: https://catalogue.ceda.ac.uk/uuid/3c324bb4ee394d0d876fe2e1db217378. In situ observational data for Blelham Tarn, Esthwaite Water, Windermere North Basin, and Windermere South Basin are available at https://eidc.ac.uk/. Data for Lakes Superior, Michigan, and Huron are available at https://www.ndbc.noaa.gov. Processed summary data for Lake Geneva, Lake Tahoe, Washington and Vättern are available at: 10.5281/zenodo.4617643.

## Code availability

The code used to produce the figures in this paper is available from the corresponding author upon request.
