# Supplementary material for: Phenological shifts in lake stratification under climate change
Source: Nat Commun. 2021 Apr 19;12:2318. doi: 10.1038/s41467-021-22657-4 (PMC8055693; doi:10.1038/s41467-021-22657-4)
Supplement: Supplementary file 1 — Supplementary Information [file 41467_2021_22657_MOESM1_ESM.pdf]

Supplementary Information

Phenological shifts in lake stratification under climate change

Woolway et al

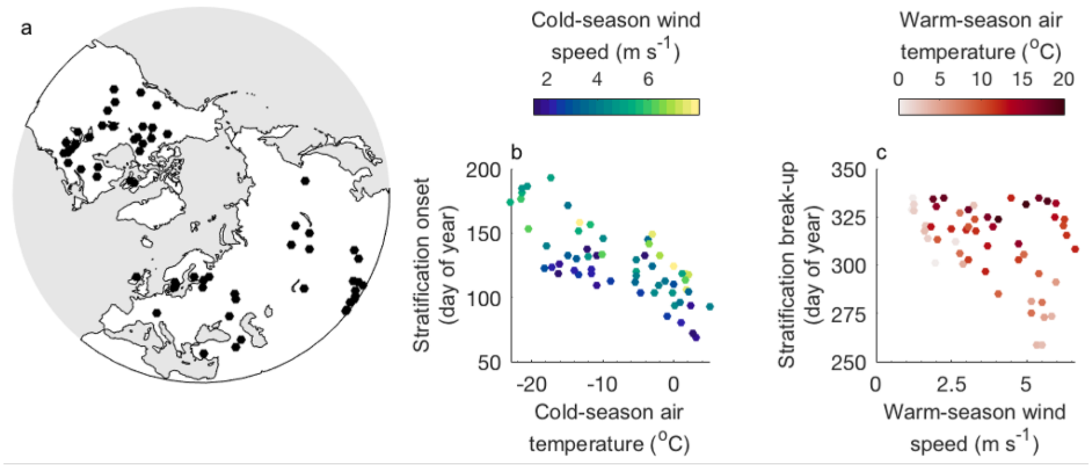

Supplementary Fig. 1 | Satellite-derived lake stratification phenology and climatic drivers. Shown for 60 studied lakes with satellite-derived lake surface temperature observations **(a)** are the historic, averaged over all years from 2007 to 2019, relationships between **(b)** stratification onset, the cold-season air temperature and wind speed, and **(c)** stratification break-up, the warm-season air temperature and wind speed. Climate data are from ERA5, extracted from the lake center pixel (see Methods).

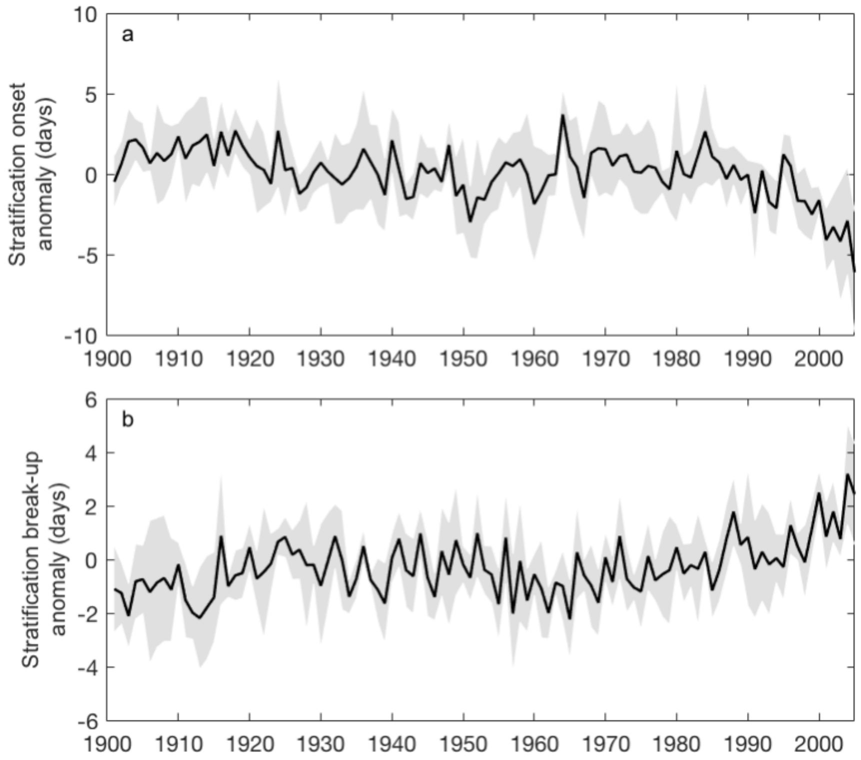

Supplementary Fig. 2 | Historic projections of lake stratification phenology. Temporal variations in **(a)** the onset and **(b)** the break-up of thermal stratification. Changes in lake stratification phenology are shown for the historic period (1901 to 2005). The thick lines show the average across all lake-climate models, and the shaded regions represent the minimum and maximum across the model ensemble. Anomalies are quoted relative to the 1970-1999 base-period average.

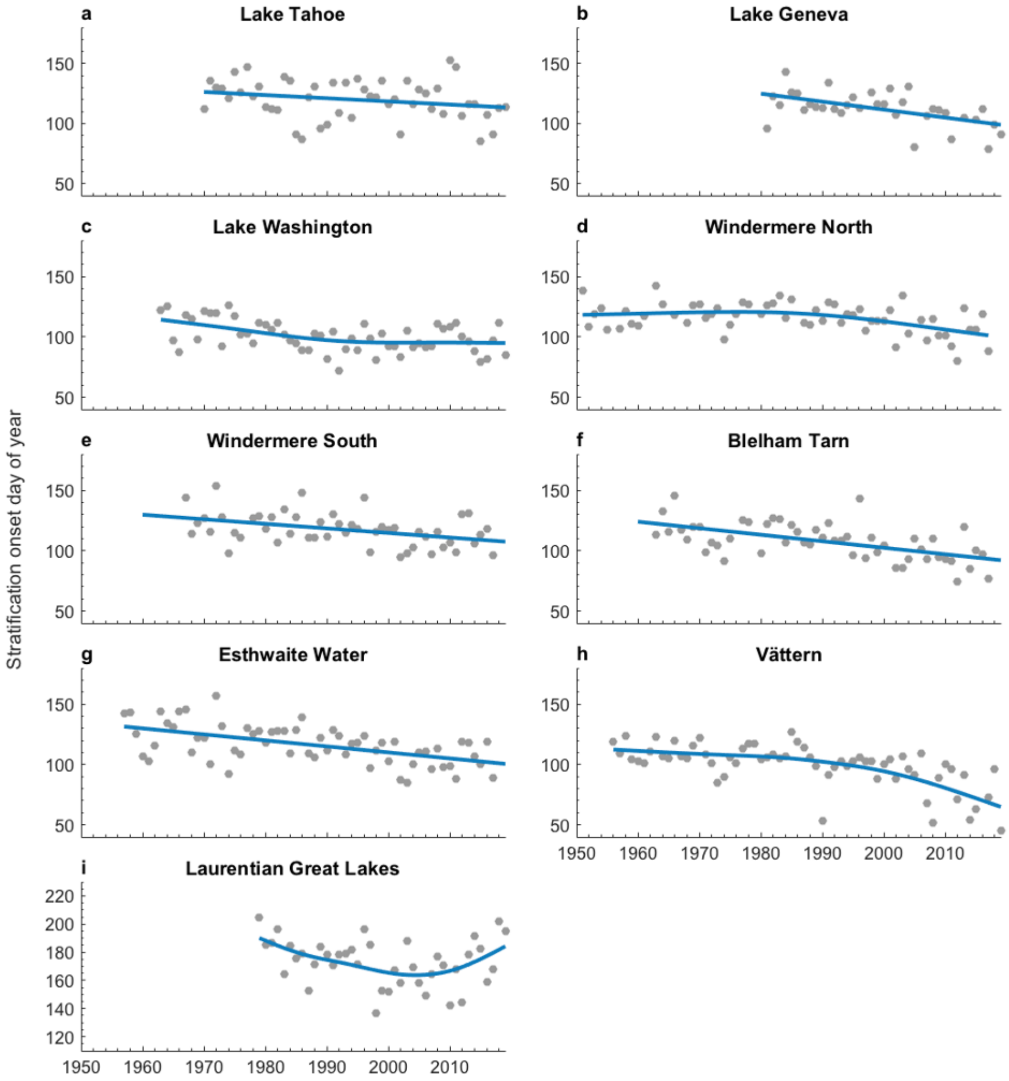

Supplementary Fig. 3 | Observed changes in lake stratification onset. Shown are the observed changes in stratification onset in some of the best monitored lakes in the Northern Hemisphere. The time period in which observational data are available differ across lakes. Long-term changes in stratification onset within the Laurentian Great Lakes (panel **i**) are averaged for all sites with available data for Lakes Superior, Michigan and Huron. The fitted line in each panel was calculated either via a linear regression model (**a, b, e, f, g**) or via a generalized additive model (**c, d, h, i**), with the optimum model selected for each lake based on the Akaike information criterion.

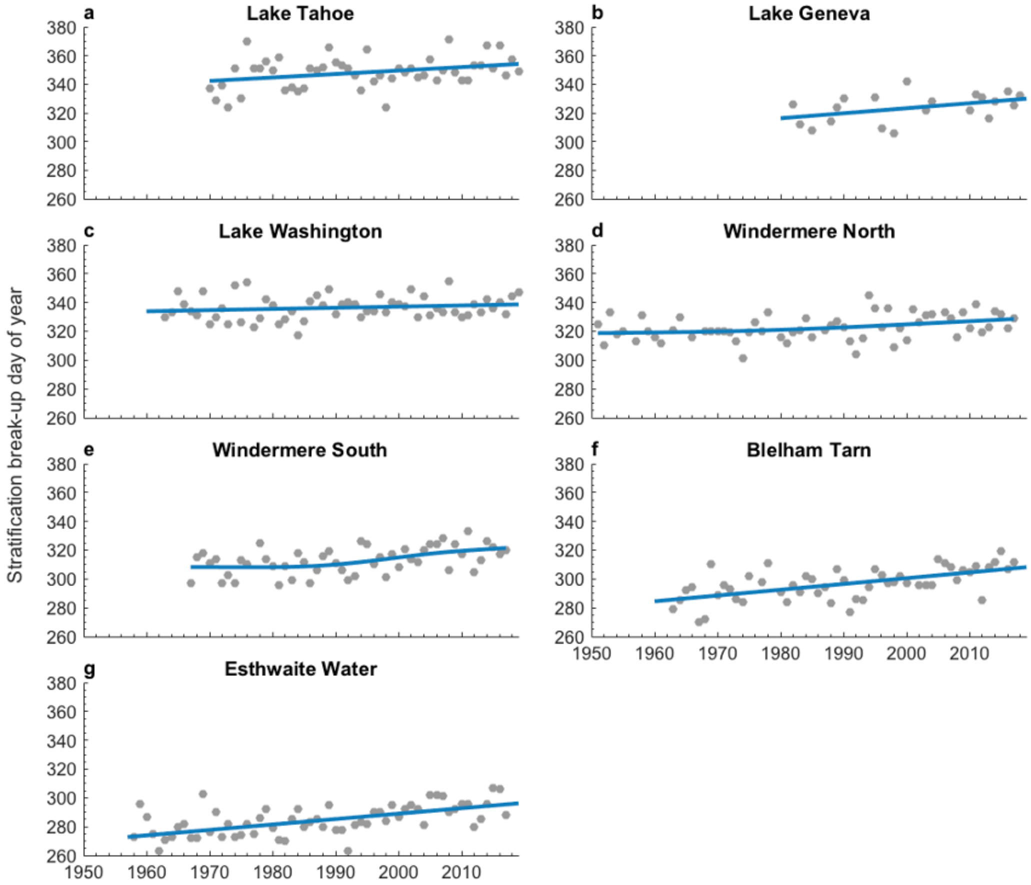

Supplementary Fig. 4 | Observed changes in lake stratification break-up. Shown are the observed changes in stratification break-up in some of the best monitored lakes in the Northern Hemisphere. The time period in which observational data are available differ across lakes. Note that stratification break-up data are not available for the Laurentian Great Lakes or Vättern (see Methods), thus panels **h** and **i** is not shown. The fitted line in each panel was calculated either via a linear regression model (**a, b, c, f, g**) or via a generalized additive model (**d, e**), with the optimum model selected for each lake based on the Akaike information criterion.

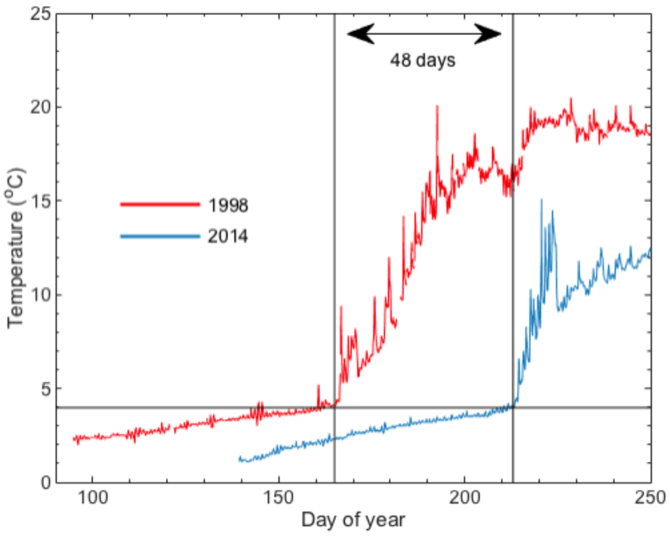

Supplementary Fig. 5 | Stratification onset in Lake Superior during two extreme years. Shown is an example of stratification onset, defined as the first day of year in which lake surface temperature exceeds 3.98 °C, in Lake Superior for two years (1998 and 2014).

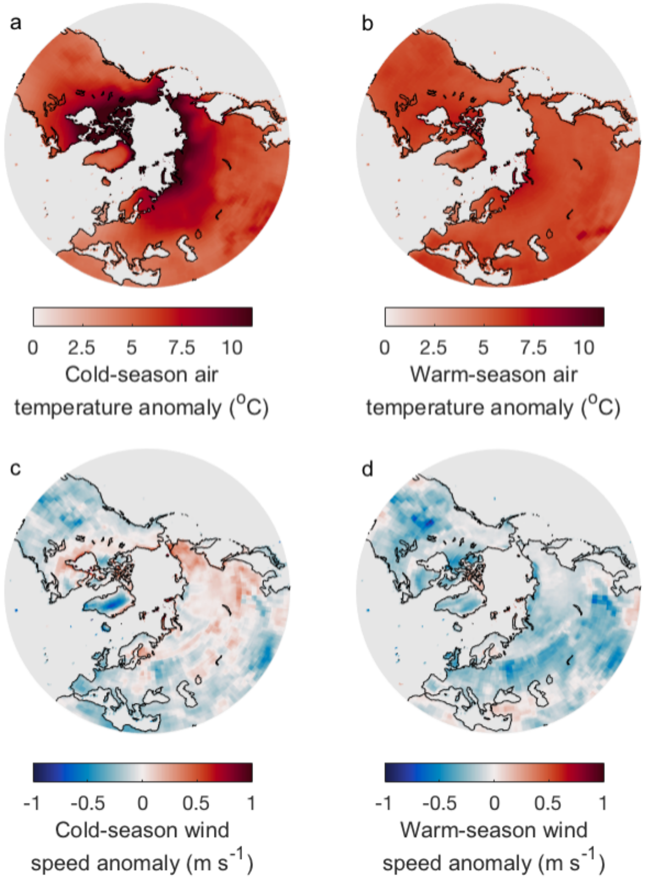

Supplementary Fig. 6 | Future simulations of air temperature and wind speed under RCP 8.5. Shown are the future, averaged over all years from 2070 to 2099, spatial patterns in **(a-b)** air temperatures and **(c-d)** near-surface wind speed averaged over the Northern Hemisphere cold (November to April) and warm (May to October) seasons. Results are shown for RCP 8.5, and represent the average across the climate model ensemble.

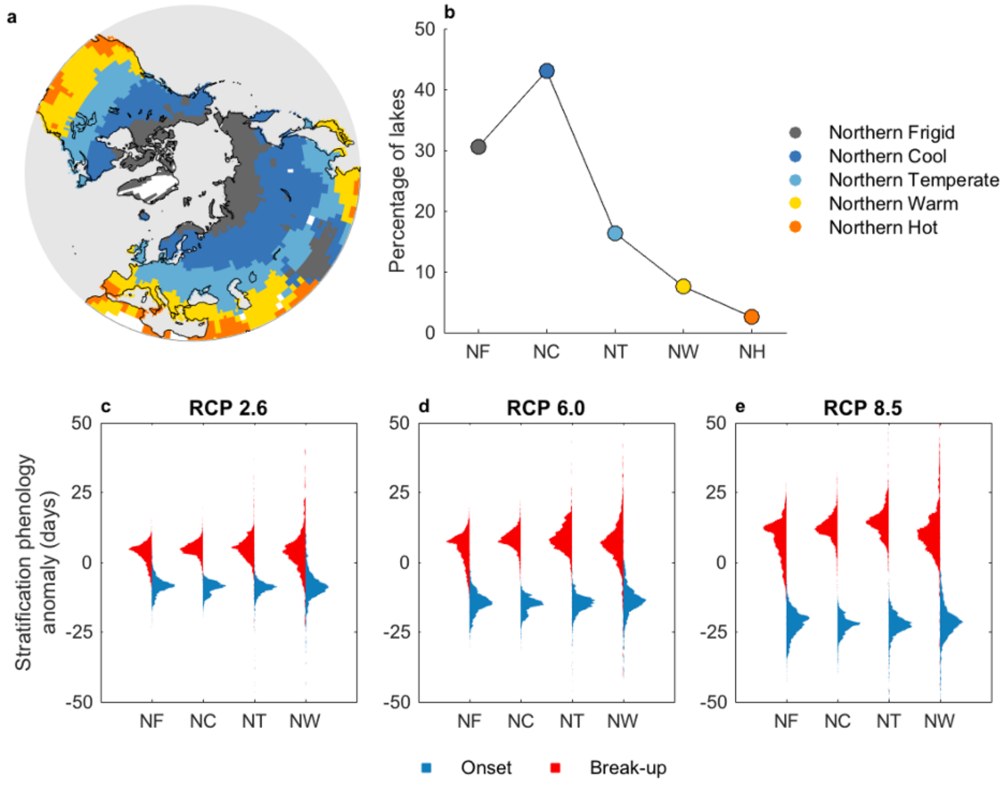

Supplementary Fig. 7 | Future projections of stratification phenology among lake thermal regions. Shown are **(a)** the lake thermal regions across the Northern Hemisphere, **(b)** the percentage of studied lakes situated within each thermal region, and **(c-e)** the change in stratification phenology by the end of the 21st century (averaged over all years from 2070 to 2099) under **(c)** RCP 2.6, **(d)** RCP 6.0, and **(e)** RCP 8.5, quoted relative to the 1970-1999 base-period average, in the four northernmost thermal regions (where the vast majority of studied lakes are situated – see panel **b**).

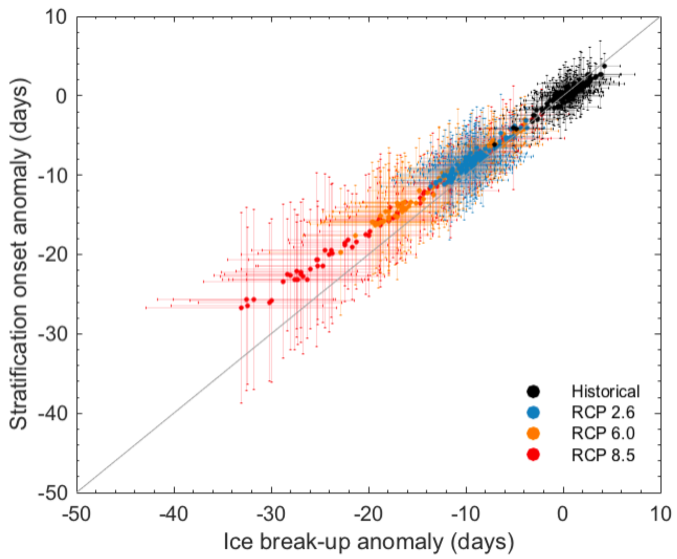

Supplementary Fig. 8 | Historic and future simulations of ice-break up and stratification onset. Shown are the relationship between the anomalies in ice break-up and the onset of thermal stratification from 1901 to 2099 under historic and future climate forcing (RCP 2.6, 6.0, 8.5). Points represent the average across all lake-climate models, and the error bars represent the standard deviation across the model ensemble. Anomalies are quoted relative to the 1970-1999 base-period average. The grey line demonstrates a 1:1 relationship between the ice break-up anomaly and the stratification onset anomaly.

Supplementary Table 1 | Correlations of climatic and in-lake drivers of lake stratification phenology. Shown are Spearman correlations between seasonal (Northern Hemisphere cold and warm season) air temperature, wind speed, lake depth, lake area and **(a)** stratification onset, and **(b)** stratification break-up. Correlations significant at $p<0.05$ are indicated in bold.

a

	Stratification onset	Cold-season air temperature	Cold-season wind speed	Lake depth	Lake area
Stratification onset	1				
Cold-Season air temperature	**-0.88**	1			
Cold-season wind speed	0.38	**-0.12**	1		
Lake depth	0.12	**-0.12**	**-0.04**	1	
Lake area	**-0.08**	0.04	**-0.06**	0.07	1

b

	Stratification break-up	Warm-season air temperature	Warm-season wind speed	Lake depth	Lake area
Stratification break-up	1				
Warm-season air temperature	0.36	1			
Warm-season wind speed	**-0.81**	**-0.34**	1		
Lake depth	0.37	**-0.14**	**-0.03**	1	
Lake area	0.13	0.11	**-0.02**	0.07	1

Supplementary Table 2 | Observed trends in lake stratification phenology. Shown are the calculated trends (via linear regression) in stratification onset and break-up across Northern Hemisphere lakes where in situ observational data are available.

Lake	Years	Stratification onset trend	Stratification break-up trend
Western Superior, USA/Canada	1981-2019	-1.8 days decade^{-1}	-
Central Superior, USA/Canada	1979-2019	-1.3 days decade^{-1}	-
Eastern Superior, USA/Canada	1980-2019	-4.1 days decade^{-1}	-
Northern Michigan, USA	1979-2019	-4.1 days decade^{-1}	-
Southern Michigan, USA	1981-2019	-3.2 days decade^{-1}	-
Northern Huron, USA/Canada	1980-2019	-7.9 days decade^{-1}	-
Southern Huron, USA/Canada	1982-2019	-2.3 days decade^{-1}	-
Tahoe, USA	1970-2019	-2.1 days decade^{-1}	+4.4 days decade^{-1}
Geneva, France/Switzerland	1981-2019	-4.4 days decade^{-1}	+5.3 days decade^{-1}
Washington, USA	1963-2019	-5.9 days decade^{-1}	+3.4 days decade^{-1}
Windermere North, UK	1951-2017	-6.4 days decade^{-1}	+7.6 days decade^{-1}
Windermere South, UK	1967-2017	-4.5 days decade^{-1}	+7.3 days decade^{-1}
Blelham Tarn, UK	1963-2017	-6.1 days decade^{-1}	+8.6 days decade^{-1}
Esthwaite Water, UK	1957-2017	-6.1 days decade^{-1}	+9.6 days decade^{-1}
Vättern, Sweden	1955-2019	-6.1 days decade^{-1}	-
